# Should We Include Connection Domain Mutations of HIV-1 Reverse Transcriptase in HIV Resistance Testing?

**DOI:** 10.1371/journal.pmed.0040346

**Published:** 2007-12-01

**Authors:** Matthias Götte

## Abstract

The author discusses a new study that found a common mutation in the connection domain of reverse transcriptase, a mutation that confers resistance to two antiretroviral drug classes.

Despite the remarkable achievements in discovery and development of antiretroviral agents for the treatment of infection with human immunodeficiency virus type 1 (HIV-1), drug resistance remains a major reason for viral rebound and treatment failure. Therefore, resistance testing has become an important tool in clinical decision making. HIV genotype testing, to look for mutations that confer drug resistance, is now widely established as the standard of care to guide treatment in the context of both primary infection and virological failure [1].

Phenotypic resistance testing involves drug susceptibility measurements in cell-based in vitro assays. Differences between wild type viral strains with no known resistance-conferring mutations and patient-derived HIV variants are usually expressed in drug concentrations required to inhibit virus replication by 50% (IC_50_). Both genotypic and phenotypic assays provide important information that helps to predict changes in drug efficacies in the context of resistance. HIV genotyping is more widespread, in part because of its lower cost.

## Limitations of Genotypic Testing

Several clinical trials showed a beneficial effect of resistance testing [2–6]. However, there are also intrinsic limitations and caveats associated with the practical use of genotyping.

First of all, the genotyping data need to be translated into predicted levels of resistance to antiretrovirals, which requires interpretation. Analysis of genotypic testing is complicated when treatment has lead to complex resistance patterns with multiple mutations [7]. The effect of a single amino acid substitution on measurable changes in drug susceptibility can sometimes markedly vary depending on the presence of other mutations or polymorphisms. Resistance or decreased susceptibility to a given drug is therefore a continuum between the two extremes of “fully sensitive” and “no antiretroviral activity,” rather than an all-or-nothing phenomenon.

**Linked Research Article**

This Perspective discusses the following new study published in *PLoS Medicine:*

Yap SH, Sheen CW, Fahey J, Zanin M, Tyssen D, et al. (2007) N348I in the connection domain of HIV-1 reverse transcriptase confers zidovudine and nevirapine resistance. PLoS Med 4(12): e335. doi:10.1371/journal.pmed.0040335

Analyzing HIV sequences from a Canadian cohort, Gilda Tachedjian and colleagues identify a common mutation in a little-studied domain of reverse transcriptase that confers resistance to two drug classes.

Drug hypersusceptibility (see Glossary) and resensitization effects add to the problems in translating genotypes into phenotypes. For instance, the M184V mutation in HIV-1 reverse transcriptase (RT) confers a high level of resistance to lamivudine (3TC) and was also shown to counteract resistance to zidovudine (AZT) in the presence of thymidine analogue–associated mutations (TAMs) [8]. Antagonistic mutations and complex resistance patterns are important reasons for apparent genotype–phenotype discordances that are often described when both genotypic and phenotypic test results are available.

Moreover, routine sequencing protocols do not cover the entire HIV genome and therefore standard genotyping systems need to be seen as surrogates of the whole genome. For practical, technical, and economical reasons, most assays involve only portions of the drug target.

Two classes of commonly used antiretroviral drugs target RT, the enzyme that copies HIV's RNA genome into DNA, leading to viral persistence in the host cell and ultimately to production of new HIV particles. Nucleoside analogue RT inhibitors (NRTIs), the first of these drug classes, are incorporated into the DNA that is being synthesized and act by preventing its further growth. The incorporated NRTI acts like a chain-terminator that prevents further addition of natural nucleotides. The second class of drugs, non-nucleoside RT inhibitors (NNRTIs), bind to RT in a manner that alters the active site and prevents it from performing DNA synthesis. The N-terminal portion of the RT enzyme constitutes the polymerase active site and includes the binding site for both NRTIs and NNRTIs. Because most NRTI and NNRTI resistance mutations are clustered around the polymerase active site and the proximate binding site for NNRTIs [9–12], routine genotypic analysis of mutations in this protein includes only about 300 or even fewer of the N-terminal amino acids, even though the entire HIV-1 RT protein encompasses 560 amino acids. However, growing evidence suggests that mutations outside the N-terminal region can likewise contribute to resistance to RT inhibitors.

**Box: Five Key Studies in the Field**

**Brehm et al., 2007** [21] Selection of mutations in the connection and RNase H domains of human immunodeficiency virus type 1 reverse transcriptase that increase resistance to 3′-azido-3′-dideoxythymidine. J Virol 81: 7852–7859. First report on the in vitro selection of AZT resistance–conferring mutations in the connection and RNase H domains.

**Nikolenko et al., 2007** [16] Mutations in the connection domain of HIV-1 reverse transcriptase increase 3′-azido-3′-deoxythymidine resistance. Proc Natl Acad Sci U S A 104: 317–322.

Shows for the first time that the C-terminal domains of HIV-1 RT can contribute to AZT resistance in the context of clinical isolates.

**Nikolenko et al., 2005** [17] Mechanism for nucleoside analog-mediated abrogation of HIV-1 replication: balance between RNase H activity and nucleotide excision. Proc Natl Acad Sci U S A 102: 2093–2098.

Provides the first evidence to suggest that diminished RNase H cleavage may increase resistance to AZT.

**Zolopa et al., 2005** [7] Accuracy, precision, and consistency of expert HIV type 1 genotype interpretation: an international comparison (The GUESS Study). Clin Infect Dis 41: 92–99. Comprehensive study on accuracy of expert HIV genotype interpretation in the context of complex mutational patterns.

**Meyer et al., 1999** [18] A mechanism of AZT resistance: an increase in nucleotide-dependent primer unblocking by mutant HIV-1 reverse transcriptase. Mol Cell 4: 35–43.

First study describing the ATP-dependent excision reaction as a mechanism for AZT resistance.

## Mutations in Connection and Ribonuclease H Domains of HIV-1 RT

The C-terminal region of RT includes the “connection” domain (289–423) and the ribonuclease H (RNase H) domain (424–560). RNase H activity is required to cleave the RNA moiety of RNA/DNA replication intermediates. G333D/E polymorphisms have been associated with dual resistance to AZT and 3TC [13,14], and Y318F has been linked to decreased susceptibility to NNRTIs [15]. More recently, Nikolenko and colleagues have shown that mutations in the C-terminal domains of HIV-1 RT can markedly increase the level of resistance to AZT, provided that these amino acid substitutions are combined with classic TAMs [16].

Nikolenko and colleagues postulated an intriguing mechanism that may help to explain the increase in AZT resistance [17]. Previous studies have shown that chain-termination with AZT, and to a lesser extent also with other NRTIs, can be reversed. HIV-1 RT is capable of removing the incorporated AZT in the presence of physiologically relevant concentrations of pyrophosphate or adenosine triphosphate (ATP) that can act as a pyrophosphate donor. TAMs containing mutant RT enzymes were shown to increase rates of the ATP-dependent excision reaction when compared with wild-type HIV-1 RT, which translates into resistance to AZT [18]. Nikolenko and colleagues then argued that AZT chain-termination might be permanent when the RT-associated RNase H activity has completely degraded the template strand. The complex of RT, the chain-terminated primer, and the cleaved template will simply dissociate under these conditions. Thus, AZT resistance could be enhanced by diminishing RNase H cleavage, which, in turn, would provide more time for the excision reaction.

Indeed, several of the newly identified mutations were shown to decrease template switching [19], which is indicative of reductions in RNase H cleavage. Most importantly, mutations E312Q, G335C/D, N348I, A360I/V, V365I, and A376S in the connection domain of HIV-1 RT were associated with increased resistance to AZT, and these mutations were identified in clinical samples of HIV-infected individuals [16]. In contrast, RNase H mutations that are seen in the clinic do not appear to contribute to AZT resistance [16,20].

Independent evidence for the role of mutations in the connection and RNase H domains in AZT resistance has been derived from in vitro selection experiments [21]. The authors reported the appearance of A371V in the connection domain and Q509L in the RNase H domain under the selective pressure of AZT. Both mutations, in conjunction with TAMs, increased resistance to AZT and also caused increased cross-resistance to 3TC and abacavir. In spite of these advances in the characterization of the C-terminal region of HIV-1 RT and its involvement in NRTI resistance, the clinical relevance of connection and RNase H mutations has remained elusive [22,23].

## A New Study of the Connection Mutation N348I

In this issue of *PLoS Medicine,* Soo-Huey Yap et al. report on a detailed clinical and biochemical characterization of the connection mutation N348I [24], which turned out to be a highly interesting mutation in many ways. Clinical samples were collected at the British Columbia Centre for Excellence in HIV/AIDS, in Vancouver, Canada. The authors compared sequences from individuals with known treatment history (*n* = 1,009) with patient samples from treatment-naïve individuals (*n* = 368) and found that N348I is highly prevalent among treatment-experienced patients. With a 12% prevalence, it ranked as the 9th most prevalent RT-associated mutation in this group of individuals.

N348I was highly associated with M184V and several different TAMs; however, analysis of 31 patient samples with no mutations present at baseline and known treatment history suggests that this mutation emerges early after initiation of antiretroviral therapy. Treatment with AZT and combined treatment with AZT and the NNRTI nevirapine (NVP) was associated with an increased detection of N348I. The authors further report that the appearance of N348I was associated with a significant increase in viral load, which was comparable with each of the individual classic TAMs at positions 219, 215, 210, 70, 67, and 41.

In vitro susceptibility studies with HIV-1 constructs that were generated by site-directed mutagenesis corroborate their findings. The authors report a moderate, 2-fold increase in AZT resistance when wild-type virus is compared with the N348I mutant variant, and an increase in AZT resistance is seen when N348I is introduced against a background of TAMs. However, the data make clear that N348I does not counteract the resensitization effects of M184V, which is also consistent with previous findings [16,20]. N348I also conferred resistance to NVP (8-fold), and this effect was amplified against a background of K103N. With the exception of rare, fitness-deficient mutations, and perhaps the effects of changes at position 181 on stavudine, this is the first example of dual resistance to NRTIs and NNRTIs.

## Possible Underlying Mechanisms

Yap et al. have also addressed the potential biochemical mechanism for how N348I confers drug resistance—they provide evidence to show that this mutation can augment levels of excision of AZT. Increased rates of excision are selectively seen on RNA/DNA substrates when TAM-containing mutant RT was compared with TAMs/N348I. Such differences are not evident on DNA/DNA substrates, which pointed to an involvement of the RT-associated RNase H activity in AZT resistance. Indeed, mutant enzymes containing N348I show significant reductions in RNase H cleavage. Thus, the biochemical data are consistent with the notion that diminished RNase H cleavage facilitates excision by delaying degradation of the template.

## Implications and Outlook

The multidisciplinary approach is a major strength of Yap and colleagues' study. Their retrospective clinical studies are in good agreement with in vitro susceptibility measurements and biochemical mechanistic analyses. The combined data provide strong evidence to show that N348I can contribute to AZT resistance. N348I may not be considered as a “secondary mutation” that solely improves the replication capacity of resistant viruses, because this mutation appears to emerge early after initiation of therapy and the diminished RNase H activity combined with elevated levels of excision provide a plausible molecular mechanism. At the same time, the decrease in AZT susceptibility is relatively small in the absence of TAMs. In this context, the authors argue that the individual effect of each of the known TAMs is likewise subtle. It is therefore tempting to categorize N348I as yet another TAM. However, there are noticeable differences between classic TAMs and N348I. The classic TAMs at positions 219, 215, 210, 41, 67, and 70 are clustered around the putative binding site of the pyrophosphate donor ATP that promotes excision of AZT. In contrast, N348I is distant from the active site and the NNRTI binding site. As the authors point out, the structural mechanism by which N348I confers resistance to AZT remains to be delineated. Moreover, the mechanism and structural basis for NNRTI resistance in association with N348I have yet to be addressed as well.

**GLOSSARY**

**C-terminal domains:** HIV-1 RT is a heterodimer that is composed of two subunits: p66 and p51. The C-terminal domains of the large p66 subunit of HIV-1 RT contain the RNase H domain that is absent in p51.

**Connection domain:** The connection domain represents the C-terminal region in p51. In p66, the “connection domain” links the N-terminal domain with the RNase H domain.

**Drug hypersusceptibility:** Mutations in HIV-1 RT that confer resistance to a given drug A can also increase susceptibility to another drug B. For instance, M184V-mediated resistance to 3TC is associated with AZT hypersusceptibility.

**G333D/E polymorphisms:** G333D/E changes in HIV-1 RT have been linked to dual resistance to AZT and 3TC. However, resistance to both drugs also emerges in the absence of G333D or G333E, and these mutations are also seen in samples isolated from untreated individuals.

**N-terminal domains:** The amino acid sequences of the N-terminal domains of p66 contain the polymerase active site.

**Resensitization effects:** Some mutations can also counteract or even reverse resistance. M184V is associated with a reversal of resistance to AZT.

**Ribonuclease H activity:** The RT-associated RNase H activity cleaves the RNA template strand when present in RNA/DNA replication intermediates.

**Thymidine analogue–associated mutations (TAMS):** Amino acid substitutions at positions 219, 215, 210, 70, 67, and 41. TAMs emerge under the selective pressure of AZT and stavudine, respectively. Two or more TAMs can often cause cross-resistance to all NRTIs.

**Y318F:** Position 318 in HIV-1 RT is located in close proximity to the NNRTI binding pocket. The Y318F mutation confers low-level resistance to the NNRTIs nevirapine and efavirenz.

In spite of these open questions, the data presented in this study help to shed light on complex resistance patterns and provide a rationale for including the N348I mutation in genotypic testing. Hachiya and colleagues have recently identified the N348I mutation in clinical isolates that showed decreased susceptibility to NVP in the absence of known NNRTI-associated mutations [25]. Thus, the inclusion of N348I, and possibly also other connection mutations, may be considered in the context of routine genotyping. Clinical studies that are designed to assess a potential benefit of including connection mutations in HIV resistance testing are required to further address this issue. It will likewise be important to gather similar information on the role of other connection mutations and possibly RNase H–associated mutations. The various locations of known C-terminal mutations in the RT enzyme suggest the involvement of different underlying mechanisms.

## Abbreviations

3TC: lamivudine
ATP: adenosine triphosphate
AZT: zidovudine
NRTI: nucleoside analogue reverse transcriptase inhibitors
NNRTI: non-nucleoside analogue reverse transcriptase inhibitors
NVP: nevirapine
RNase H: ribonuclease H
RT: reverse transcriptase
TAM: thymidine analogue–associated mutation
